# A tribute to Isao Karube (1942–2020) and his influence on sensor science

**DOI:** 10.1007/s00216-020-02946-5

**Published:** 2020-09-18

**Authors:** Frieder W. Scheller, Rolf Schmid

**Affiliations:** 1 Institute for Analytical Biochemistry, University of Potsdam, Karl-Liebknecht-Str., 14476 Potsdam, Germany; 2 Institute of Technical Biochemistry, University of Stuttgart, Allmandring 31, 70569 Stuttgart, Germany

**Keywords:** Karube, Japan, Biosensors, Lifetime achievements

**Fig. 1:**
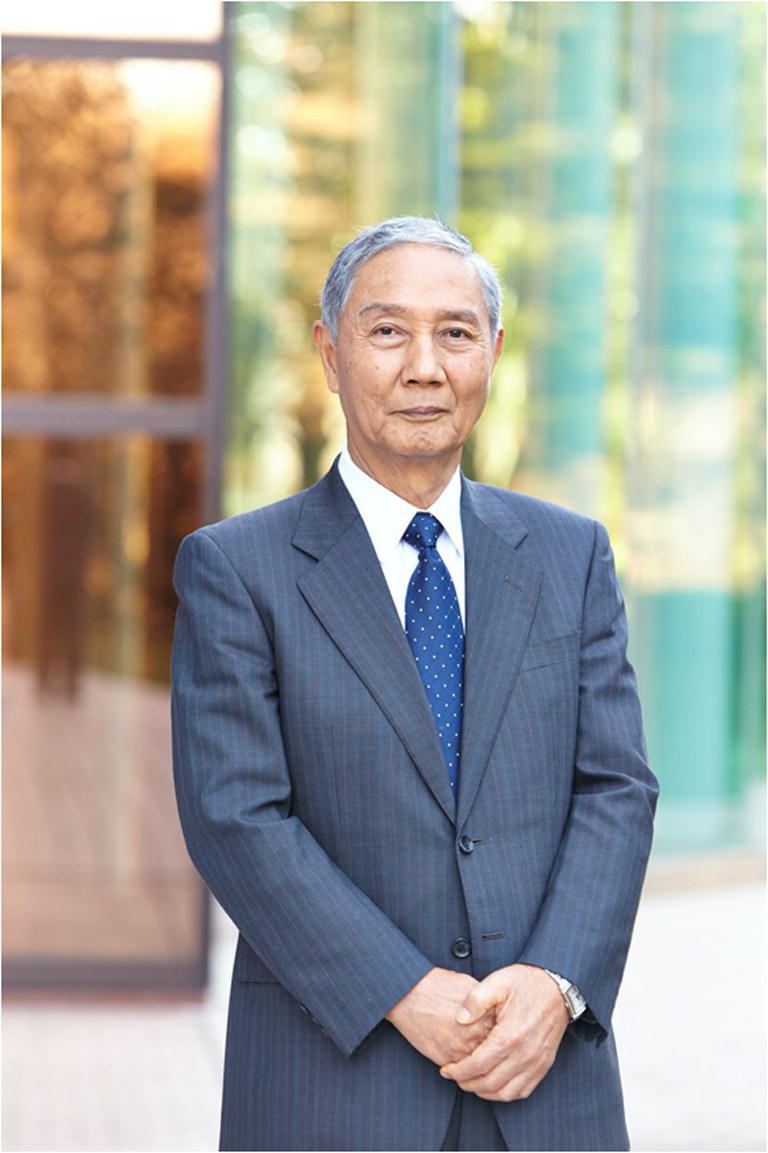
Isao Karube; © Tokyo University of Technology, with permission

We report with great sadness that Japan’s “Mr. Biosensor”, Professor Isao Karube, has passed away in Yokohama aged 78 (Fig. 1).

Karube’s love for and excellence in science has inspired hundreds of young scientists in his laboratory and thousands of scientists around the world, many of which belong to *ABC*’s authors, peer reviewers and readers.

This journal devoted a special issue to Karube on the occasion of his 60th birthday in January 2002 [1]. Fourteen reports highlighted innovative molecular recognition elements for bioanalysis including engineered Fab fragments, new phosphate binding proteins, variants of acetylcholine esterases, molecularly imprinted polymers, aptamers, electrochemical enzyme sensors, and optical immunosensors. In all these areas, the Karube group initiated many new concepts, which are documented in some 600 publications and hundreds of patent publications and pursued by over 50 professors who have emanated from his school at the Research Center for Advanced Science and Technology (RCAST) of the University of Tokyo, as documented in a comprehensive obituary [2].

We, long-standing colleagues and close friends of Isao Karube, wish to pay tribute to his lifetime achievements by emphasizing his extraordinary success in the translation of science to industry and politics and even to the mind of the general public.

Karube was not a shy, introverted scientist. He was a polite, but outspoken, man, and his interests clearly went beyond science and teaching. In his academic environment, he soon became involved in science policy, e. g., through the Life Science Committee of the Ministry of Culture (Monbusho) and through various advisory bodies of the Ministry of Trade and Industry (MITI) whose primary objectives were to foster scientific innovation in Japan’s industries. As a consequence of such programs, many doctoral students in Karube’s group came from industry. It is not uncommon in Japanese industry to send talented young employees back to university for training within a master course or even a doctorate. This is usually done with the objective to introduce new technologies to the company. In Karube’s lab, more than 80 researchers dispatched from industry completed their master or doctoral theses, putting him in the enviable situation that the latest technical devices were at his disposition: ultrasound monitors, image analyzers, telemetric systems, lasers, or laboratory robots. Employees of the watch company Seiko investigated in Karube’s lab how to use biomolecules on quartz devices as piezoelectric sensors. Junior researchers from Fujitsu and NEC brought the latest electronic components from their companies to improve the precision and response time of amperometric glucose microbiosensors. Ajinomoto sent a researcher to develop on-line fermentation control of glutamic acid and lysine. When Karube patented a “toilet sensor,” profiling human urine for analytes such as glucose which might indicate a metabolic disease, the pertinent patent was immediately transferred to Japan’s major toilet-producing company Toto. When his group developed the first environmental biosensor based on immobilized microorganisms which measure biological oxygen demand (BOD) within a few minutes—a breakthrough in waste water technology as the standard test format required 5 days—commercial devices soon followed. The impact of these early works on microbial biosensors was recently reviewed in *ABC* [3]. The group’s “fish freshness sensor” measuring xanthine and volatile amines was among the first multi-enzyme electrodes and found commercial application in a paper strip version. A substantial number of studies following up on these innovations have been published in *ABC* [4].

It is quite obvious that policy makers quickly recognized Karube’s talents. As one of us (RS) could observe through a 3-month sabbatical at his school in 1991, he spent many hours every week at the offices of the Ministry of Trade and Industry (MITI), both as an adviser for national issues of science policy and, as he had spent some postdoctoral years at the University of Illinois and was both adept in American English and the American mindset, as a member of the “trade friction committee” which modulated US-Japan ambiguities in trade. As a consequence, quite a few of the MITI programs of the 1990s bear traces of Karube’s original ideas. At a local level, several governors of Japanese prefectures requested him to become their science adviser on environmental issues such as pollution monitoring or combatting seaweed pests. In this context, he was instrumental in establishing an International Center for Environmental Technology Transfer (ICETT) in Yokkaichi, Mie Prefecture.

A demanding university position and an inspiring political agenda may seem sufficient to fill a busy man’s schedule. However, Isao Karube energetically entered into a third field: the scientific education of the general public. His 20 or so booklets, some of them in manga style, bear titles such as “Bionics,” “Environmental biotech,” “Bioremediation,” or “The wonders of super-power enzymes,” and also reach out for infotainment on issues such as “How to increase your creativity,” “How to stay young up to age 120,” or “Biosociety - the chatter of life creates the technologies of the future.” Still on sale, they address the business manager on his long commuter rides between home and office and have achieved surprising circulation numbers. In the 1990s, they laid the foundation for Karube’s television shows where he interviewed leading scientists on prime time.

The retirement age at Japan’s State Universities such as the University of Tokyo used to be 60, and Karube’s retirement was celebrated accordingly on March 6, 2002, with an International Conference on Biotechnology and Bioelectronics in the splendid environment of Tokyo’s Hotel Okura. Key contributions of Karube’s group were reviewed at this time in a comprehensive publication in *ABC* [5]. At this occasion, we also learnt about the next step in Isao Karube’s life: he would become president of one of Japan’s biggest private universities, the Tokyo University of Technology, located in Hachioji, a suburb of Tokyo.

As could be anticipated, Karube developed his new school energetically (again, one of us (RS) spent 1 month there in 2008). The Schools of Computer Science and Media Sciences were strengthened, and a new School of Bionics was established in 2003 under his leadership. Due to his excellent network, Karube managed to introduce a government technology center into his campus—a first among Japan’s private universities. He was able to recruit leading experts as teachers for the new schools. Among his special concerns was to enhance the global competence of his students through international exchange programs and language classes provided by native English speakers. In 2010, Karube established a new campus located at Kamata, overlooking Tokyo’s busy Haneda International Airport and very close to Kanagawa prefecture where much of Eastern Japan’s industry is located, in search of university graduates. It hosts two new schools: The School of Design and the School of Health Sciences.

In early 2020, after an extremely busy and successful life, Isao Karube has passed away, much too early to reap the benefits of a peaceful retirement. We, his foreign colleagues, will honor his memory as a distinguished scientist, as an inspiring communicator, and as a true friend, extending our condolences and sympathies to his family.
